# Need for improving immunization status and preventive care in diabetes mellitus patients

**DOI:** 10.1007/s00508-022-02080-5

**Published:** 2022-09-22

**Authors:** Teresa Gisinger, Alexandra Kautzky-Willer, Michael Leutner

**Affiliations:** 1 Clinical Division of Endocrinology and Metabolism, Department of Internal Medicine III, Medical University of Vienna, Waehringer Guertel 18–20, 1090 Vienna, Austria; 2 Gender Institute, 3571 Gars am Kamp, Austria

**Keywords:** Diabetes mellitus, Preventive care medicine, Immunization, Public Health, Disease Prevention

## Abstract

**Background:**

The incidence and the comorbidities, such as infectious diseases (e.g. pneumonia or influenza) of diabetes mellitus are increasing. Therefore, the purpose of this study is to investigate immunization status and preventive care in diabetes mellitus patients.

**Methods:**

Two groups from the Austrian health interview survey 2014 were identified, a cohort of diabetes mellitus (DM) individuals (*n* = 678) and a non-diabetes mellitus (non-DM) cohort (*n* = 15,093). The frequencies of doctors’ visits, preventive care and immunization status were compared. Furthermore, the study population was divided by age (> 50 years, < 50 years) and differences between > 50 years old DM with < 50 years old DM and the > 50 years old DM and > 50 years old Non-DM cohort were investigated.

**Results:**

In the DM cohort a higher frequency of influenza immunization (13.3% vs. 7.1%, *p* < 0.001), doctor visits (89.4% vs. 75.4%, *p* < 0.001), and preventive care, such as colonoscopy (11.2% vs. 6.8%, *p* < 0.001) and hemoccult tests (32.6% vs. 22.1%, *p* < 0.001) was observed. Even though older DM individuals have a higher risk for complications, the > 50 years DM cohort has similar frequencies of colonoscopy, hemoccult test and immunization against influenza and TBE (tick-borne encephalitis) compared to > 50 years Non-DM. Although the > 50 years old DM cohort had a higher frequency of doctors’ visits, they still had lower frequencies of mammography and dentists’ visits compared to > 50 years old Non-DM. In comparison to the < 50 years old DM cohort, the > 50 years DM cohort was related to lower intact immunization status of tetanus, diphtheria, Polio and TBE. Still a higher frequency of intact immunization of pneumococcus, influenza and doctors’ visits in the > 50 years old DM cohort compared to the < 50 years old DM cohort can be reported.

**Conclusion:**

Preventive care and immunization status in the DM cohort just differ slightly from the general cohort but still should be improved.

**Supplementary Information:**

The online version of this article (10.1007/s00508-022-02080-5) contains supplementary material, which is available to authorized users.

## Introduction

Diabetes mellitus is a global burden with massive economic costs. Around 12% of global expenditure on health is assigned to the care of individuals with diabetes mellitus (DM) and its related complications [1]. In 2020, 463 million adults worldwide suffered from diabetes mellitus and studies investigated that the number of diabetes mellitus patients will rise to 471 million until 2030 [1]. Furthermore, in 2013 the Global Burden of Disease Study listed diabetes mellitus as the ninth major cause of reduced life expectancy [2]. In 2010, diabetes mellitus made 6.8% of the global mortality and therefore led to 3.96 million deaths in adults aged 20–79 years [3]. Even though the mortality rate of DM individuals is decreasing, this specific cohort is still at higher risk when compared to non-diabetes mellitus (non-DM) individuals [4]. The cause of diabetes mellitus being a global burden is its morbidity and mortality, mostly resulting from its complications [1, 2], namely microvascular and macrovascular complications [5, 6]. With the duration of diabetes mellitus the incidence of these complications is rising and also reduced life expectancy occurs in most of the cases [6]. Patients with diabetes and comorbidities, such as hypertension and dyslipidemia had more diabetes complications [7]. Further, it has been investigated that diabetes mellitus affects the incidence of cancer and therefore especially preventive care is important in this cohort [8]. Hyperglycemia in cancer patients leads to increased rates of adverse events and outcomes [9]. In addition, diabetes mellitus can alter the immune system and therefore increase the risk of infections. For example, an Austrian study investigated higher risk for pneumonia in a cohort with diabetes mellitus [10]. In particular the high blood glucose levels in DM individuals alters the immune system and impairs the immune response [11]. Previous studies investigated that an excess of glucose in systemic circulation can cause an increase in reactive oxygen species (ROS) and the proinflammatory cytokines interleukin 1 (IL-1) and interleukin 6 (IL‑6) [12, 13].

In the context of the increased risk of complications in individuals with DM, especially data about the preventive care status are sparse. Hence the aim of the present study was to investigate the preventive care and immunization status in the high-risk population of individuals with DM.

## Patients, material and methods

### Study design

This retrospective study was conducted in 2014 and used self-administered paper questionnaires and computer-assisted telephone interviewing for data collection.

### Source of data

The data for this study were provided from a large, national public health survey, namely the Austrian Health Interview Survey (AT-HIS *n* = 15,771). It was part of the European Health Interview Survey (E-HIS). AT-HIS included individuals over the age of 15 years, who are living in Austria and were randomly chosen and asked about their health, their lifestyle and their utilization of the healthcare system. The participants were recruited within the framework of the Austrian sample census in 2013.

### Measure

This dataset includes information about demographic, lifestyle and social variables as well as overall health, chronic conditions and healthcare utilization. It includes comorbidities such as diabetes mellitus, arterial hypertension, chronic kidney disease, liver cirrhosis, myocardial infarction, angina pectoris or coronary heart disease. Further it records the frequency of using preventive care, such as Papanicolaou smear (PAP smear test), mammography, test for occult blood in feces and colonoscopy. AT-HIS also captures the immunization status and the frequency of doctors’ visits of the study population. In AT-HIS age was collected in age groups: therefore, the first age group considers individuals in the age between 15–19 years, the second group consisted of DM individuals 20–24 years old, the third of 25–29 years and so on until the age group 95 years and older. The group of cardiovascular diseases is defined by a positive history of myocardial infarction, angina pectoris and/or coronary heart disease. The variable stroke consists of individuals suffering from stroke or stroke consequences. Sleep disorder is specified as having trouble to fall asleep or being intensely tired on nearly every day or more than half of the time.

### Statistical analysis

At first, data dictionary was scanned for variables suitable for analysis. The variables were grouped into baseline characteristics, and health and preventive care as doctors’ visits, preventive care screening and immunization status. Then two cohorts were formed, one out of diabetes mellitus (DM) individuals (*n* = 678) and one out of non-diabetes mellitus (non-DM) individuals (*n* = 15,093). Frequencies for every variable of both cohorts were investigated. Further the interaction between diabetes mellitus and all variables was explored to determine if the impact of the variables differed for DM and non-DM individuals by a linear and logistic regression model. Moreover, both cohorts were separated by age. At first individuals with DM over the age of 50 years and under the age of 50 years were compared. The frequencies for every variable were analyzed and the interaction of age on all variables was explored by a linear and logistic regression model. A group of individuals over 50 years with diabetes mellitus was compared to a group over 50 years without diabetes mellitus. Also, for the groups divided by age the frequencies of all variables and the interaction by linear and logistic regression were investigated. For all analyses statistical significance was defined with a *p*-value < 0.05. Analyses were carried out by R version 4.0.0 (Robert Gentleman and Ross Ihaka—also known as “R & R” of the Statistics Department of the University of Auckland, Auckland, New Zealand).

## Results

### Baseline characteristics

In total the study included 15,771 individuals in AT-HIS, of which 678 were diagnosed with diabetes mellitus. The baseline characteristics of the study population are presented in Table 1. Individuals with DM are significantly older and were characterized by a higher BMI. Out of the DM cohort 52.7% were men and 47.3% were women. Out of the non-DM individuals 43.9% were men and 56.1% were women. Table 1 presents that DM individuals were at higher risk of being diagnosed with several comorbidities, including cardiovascular disease, arterial hypertension, stroke, chronic kidney disease, depression or sleeping disorders. In Table 1 the baseline characteristics for the cohort with DM and without DM were analyzed. Next in Table 1 individuals over 50 years with and without diabetes mellitus were observed. Comparing these groups similar results when comparing DM versus non-DM individuals were seen.

**Table 1:** Frequencies of basic characteristics for the DM and non-DM cohort and all *p*-values for the linear regression analyses. Frequencies of basic characteristics for the cohort with and without DM and all *p*-values for the linear regression analyses. Frequencies or mean value and standard deviation of basic characteristics for the over 50 years old DM and over 50 years old Non-DM cohorts and all *p*-values for the linear regression analyses

Variable name	DM (*n* = 678)	Non-DM (*n* = 15,093)	*p*-value	Over 50 years DM (*n* = 592)	Over 50 years Non-DM (*n* = 6689)	*p*-value
*Sex* Men Women	– 357 (52.7%) 321 (47.3%)	– 6628 (43.9%) 8465 (56.1%)	< 0.001	– 313 (52.9%) 279 (47.1%)	– 2918 (43.6%) 3771 (56.4%)	< 0.001
Mean age groups (years)	60–64	45–49	< 0.001	–	–	–
BMI (kg/m^2^)	29.5 ± 5.5	25.1 ± 4.3	< 0.001	29.5 ± 5.3	26.1 ± 4.2	< 0.001
CVD (*n* = 133)	36 (5.3%)	97 (0.6%)	< 0.001	35 (5.9%)	83 (1.2%)	< 0.001
Arterial hypertension (*n* = 3160)	422 (62.2%)	2738 (18.1%)	< 0.001	393 (66.4%)	2179 (32.3%)	< 0.001
Stroke (*n* = 116)	22 (3.2%)	94 (0.6%)	< 0.001	21 (3.5%)	79 (1.2%)	< 0.001
Liver cirrhosis (*n* = 37)	5 (0.7%)	32 (0.2%)	0.001	5 (0.8%)	19 (0.3%)	0.05
CKD (*n* = 198)	40 (5.9%)	158 (1.0%)	< 0.001	40 (6.8%)	107 (1.6%)	< 0.001
Depression (*n* = 1053)	96 (14.2%)	957 (6.3%)	< 0.001	83 (14.0%)	578 (8.6%)	< 0.001
Sleep disorder (*n* = 1912)	155 (22.9%)	1757 (11.6%)	< 0.001	135 (22.8%)	1064 (15.9%)	< 0.001

*DM* diabetes mellitus, *Non-DM* non-diabetes mellitus, *BMI* body mass index, *CVD* cardiovascular disease, *CKD* chronic kidney disease

### Immunization

Looking at immunization a higher immunization status coverage against influenza (13.3% vs. 7.1%, p < 0.001) and pneumococcus in the DM cohort compared to the non-DM cohort (13.8% vs. 9.9%, *p* < 0.001) was found. The non-DM cohort was more likely to have an intact immunization status against tetanus (80.0% vs. 70.1%, *p* < 0.001), diphtheria (57.7% vs. 39.7%, *p* < 0.001), polio (52.0% vs. 37.0%, *p* < 0.001) and TBE (69.3% vs. 63.0%, *p* < 0.001, Table 2). Comparing the > 50 years old DM cohort to the > 50 years old non-DM cohort no difference in influenza and TBE immunization can be found. The higher frequency of pneumococcus immunization within the DM cohort compared to the non-DM cohort in the population over 50 years is still present (9.5% vs. 5.0%, *p* < 0.001), as well as higher frequency of tetanus (73.5% vs. 67.7%, *p* = 0.01), diphtheria (46.7% vs. 36.1%, *p* < 0.001) and polio (44.0% vs. 34.6%, *p* < 0.001) in the Non-DM cohort compared to the DM cohort in the population over the age of 50 years. Lastly, the DM cohort was split into > 50 years and < 50 years (Supplementary Table 2). Compared to the < 50 years DM cohort, the > 50 years DM cohort had a lower frequency of tetanus (67.7% vs. 86.0%, *p* < 0.001), diphtheria (36.1% vs. 64.0%, *p* < 0.001), polio (34.6% vs. 53.5%, *p* < 0.001) and TBE immunization (66.9% vs. 67.4%, *p* < 0.001); however, the frequency of influenza (13.3% vs. 7.1%, *p* < 0.001) and pneumococcus immunization (13.8% vs. 9.9%, *p* < 0.001) was higher in the > 50 years DM cohort.

**Table 2:** Frequencies of immunization for the DM and non-DM cohort and all *p*-values for the linear regression analyses. Frequencies of immunization for the over 50 years DM and the over 50 years Non-DM cohort and all *p*-values for the linear regression analyses

Variable name	DM (*n* = 678)	Non-DM (*n* = 15,093)	*p*-value	Over 50 years DM (*n* = 592)	Over 50 years Non-DM (*n* = 6689)	*p*-value
Intact immunization against tetanus	475 (70.1%)	11,917 (80.0%)	< 0.001	401 (67.7%)	4915 (73.5%)	0.01
Intact immunization against diphtheria	269 (39.7%)	8676 (57.5%)	< 0.001	214 (36.1%)	3126 (46.7%)	< 0.001
Intact immunization against polio	251 (37.0%)	7847 (52.0%)	< 0.001	205 (34.6%)	2944 (44.0%)	< 0.001
Intact immunization against pneumococcus	56 (13.8%)	333 (9.9%)	< 0.001	56 (9.5%)	333 (5.0%)	< 0.001
Intact immunization against influenza	90 (13.3%)	1072 (7.1%)	< 0.001	84 (14.2%)	741 (11.1%)	0.05
Intact immunization against TBE	427 (63.0%)	10,462 (69.3%)	< 0.001	396 (66.9%)	4377 (65.4%)	> 1

*DM* diabetes mellitus, *Non-DM* non-diabetes mellitus, *TBE* tick-boren encephalitis

### Doctor visits and hospital stays

The DM individuals more commonly visited the general practitioner and specialist doctors (e.g. endocrinologist, cardiologist) in comparison to non-DM individuals (89.4% vs. 75.4%, 74.5% vs. 64.0%, *p* < 0.001). The non-DM group had a higher frequency of dentist visits compared to the DM group (48.6% vs. 38.8%, *p* < 0.001) (Table 3). In the > 50 years old DM cohort a lower frequency of dentist visits was documented compared to the > 50 years old Non-DM cohort (Table 3). The individuals over 50 years of age within the DM cohort had a higher frequency of being hospitalized in the last 12 months (32.1% vs. 16.3%, *p* < 0.001), but had a lower frequency of visiting the dentist compared to the DM individuals under 50 years of age (38.0 vs. 44.2%, *p* = 0.01).

**Table 3:** Frequencies of doctor visits for the DM and non-DM cohort and all *p*-values for the linear regression analyses. Frequencies of doctor visits for the over 50 years DM and the over 50 years Non-DM cohort and all *p*-values for the linear regression analyses

Variable name	DM (*n* = 678)	Non-DM (*n* = 15,093)	*p*-value	Over 50 years DM (*n* = 592)	Over 50 years Non-DM (*n* = 6689)	*p*-value
Hospitalized in the last 12 months	204 (30.1%)	2089 (13.8%)	< 0.001	190 (32.1%)	1265 (18.9%)	< 0.001
*Dentist* In the last 6 months in the last 6–12 months over 12 months ago	– 263 (38.8%) 146 (21.5%) 269 (43.7%)	– 7329 (48.6%) 4140 (27.4%) 3624 (24.0%)	< 0.001	– 225 (38.0%) 121 (20.4%) 246 (41.5%)	– 3152 (47.1%) 1679 (25.1%) 1858 (27.8%)	< 0.001
*General practitioner* In the last 12 months over 12 months ago never	– 606 (89.4%) 69 (10.2%) 3 (0.4%)	– 11,374 (75.4%) 3605 (23.9%) 114 (0.8%)	< 0.001	– 534 (90.2%) 55 (9.3%) 3 (0.5%)	– 5129 (76.7%) 1505 (22.5%) 55 (0.8%)	< 0.001
*Specialist doctor* In the last 12 months over 12 months ago never	– 505 (74.5%) 163 (24.0%) 10 (1.5%)	– 9655 (64.0%) 5113 (33.9%) 325 (2.2%)	< 0.001	– 436 (73.6%) 146 (24.7%) 10 (1.7%)	– 4460 (66.7%) 2102 (31.4%) 127 (1.9%)	0.01

### Preventive care

In comparison to the non-DM cohort, the total DM-cohort was more likely to use preventive care, such as hemoccult test (32.6% vs. 22.1%, *p* < 0.001), mammography (36.4% vs. 31.0%, *p* < 0.001) and colonoscopy (11.2% vs. 6.8%, *p* < 0.001). The DM individuals also had a higher frequency of laboratory measurements, such as blood glucose measurements (92.2% vs. 57.2%, *p* < 0.001) and blood cholesterol (90.0% vs. 55.8%, *p* < 0.001). In the supplementary Table 1 all frequencies and *p*-values can be found. Next the population older than 50 years was observed. In the > 50 years old DM cohort a higher likelihood of blood glucose measurements (91.2% vs. 69.1%, *p* < 0.001), blood cholesterol (90.0% vs. 68.7%, *p* < 0.001) and blood pressure measurements (91.9% vs. 77.1%, *p* < 0.001) could be observed, as well as a lower frequency of mammography compared to the > 50 years old Non-DM cohort (38.4% vs. 43.0%, *p* = 0.01). In supplementary Table 2 the DM cohort was split and compared according to age > 50 years old and <50 years old. Hence, the > 50 years old DM cohort had a higher frequency of colonoscopy (11.8% vs. 7.0%, *p* < 0.001) and hemoccult test compared to the < 50 years old DM cohort (34.1% vs. 22.1%, *p* < 0.001).

## Discussion

The present study could observe that DM individuals who are characterized by a higher frequency of comorbidities, more commonly visited a general practitioner and specialist doctors and also had a higher frequency of preventive care testing such as colonoscopy, mammography, PAP smear and hemoccult test. Additionally, they were more commonly immunized against influenza when compared to non-DM controls. Similar results concerning being hospitalized in the last 12 months, influenza immunization status and frequency of hemoccult test and colonoscopy in the > 50 years old DM cohort compared to the < 50 years old DM cohort could be found. Further comparing the > 50 years old DM to the > 50 years old non-DM cohort, similar trends as in the non-age stratified comparison were investigated. The only discrepancies are no difference of frequency of influenza and TBE immunization, colonoscopy, hemoccult tests and PAP smears. Differences investigated between women and men with DM were a higher number of cardiovascular disease in men and more sleep disorders in women. These findings are in line with the reports of AT-HIS 2007 [14].

As mentioned before the high blood glucose in diabetes mellitus alters the immune system and impairs the immune response [11]. The present study investigated that individuals with DM are more likely to have a higher influenza immunization coverage. Also, a Swiss study found a high number of annual flu shots in DM individuals [16]. The higher frequency of influenza shots in the DM population could result from more frequent visits to the general practitioner. Current literature shows that individuals with DM have a higher incidence for influenza and pneumonia and further are at higher risk for influenza-mediated and pneumonia-related morbidity and mortality [10, 15]. Due to the higher influenza-related morbidity and mortality in individuals with diabetes mellitus the general practitioner could be more likely to suggest the annual flu shot to DM individuals. In the age-stratified analysis, a higher frequency of influenza immunization within the > 50 years old DM cohort compared to the < 50 years old DM cohort can be found; however, the difference in intact influenza immunization cannot be found in the comparison of the > 50 years old DM to the > 50 years old Non-DM populations. Even though the immune system of DM individuals is weaker compared to healthy individuals, especially in older patients. Therefore, all DM individuals should be vaccinated against influenza. Next the intact immunization of other infections such as tetanus, diphtheria, polio and TBE is still statistically significantly lower in the DM cohort compared to the non-DM cohort. In the present study an intact immunization of pneumococcus in 13.8% of the DM cohort could be observed. In comparison, another study could show that around 39% of DM individuals received a pneumococcal vaccination in 2007 [17]. Still, in order to protect people with DM the target should be to improve the immunization rate against all infections.

Concerning doctors’ visits, the results on visiting general practitioners of DM individuals (89.4%) are in line with recently published data of a Swiss study group, who demonstrated that around 93.4% of DM individuals attended their general practitioner annualy [16]. In the Swiss study, around two thirds of the DM individuals visited a diabetologist [16]. Looking at our results, 74.5% of the DM cohort went to a specialist doctor in the last year. The frequency of attending a specialist doctor in the present study is higher than in Switzerland, as this study includes all specialties compared to only diabetologists in Switzerland. In general, the higher amount of general practitioners and specialist doctors’ visits could result from the higher comorbidities found in the DM cohort. Therefore, the individuals of the DM group could have more check-ups and visits related to complications from the underlying disease or comorbidities and visit a doctors’ office more often. Next in 2014 an US study investigated the differences in annual dentist visits between a DM and a non-DM group. They observed that around 61.4% of the DM group went to the dentist in the last year compared to 66.5% in the non-DM group [18]. In their study similar trends could be found. In our study around 38.8% of the DM cohort visited the dentist in the last 6 months compared to 48.6% of the non-DM cohort. Even though periodontal disease is known to be more frequent in diabetes mellitus patients the authors could observe a lower amount of annual dentist visits in the DM group. Possible mechanisms linking periodontal disease to diabetes mellitus include elevated systemic levels of pro-inflammatory cytokines, especially interleukin‑1, interleukin‑6 and tumor necrosis factor alpha [19]. Furthermore, studies also claim that periodontal disease are related to the development of diabetes mellitus [19]. Moreover, periodontal disease increases with age [20]. Still, the > 50 years old DM cohort was less likely to visit the dentist compared to the > 50 years old Non-DM cohort. This finding could result from the low awareness of the impact of diabetes mellitus on oral health in the DM cohort [21]. Further, the insurance companies in Austria do not cover every treatment carried out by the dentist. Therefore, socioeconomic situation of DM individuals could also affect the frequency of visiting the dentist. Interestingly the present study observed higher amounts of preventive actions in the DM cohort compared to the non-DM cohort, except for PAP smears and mammography. Also, looking at blood pressure measurements and laboratory measurements for the analysis of blood glucose and blood cholesterol levels, there was a higher frequency in the DM cohort. Similar trends can be observed in a Swiss study, showing that 96% of the DM group had a blood pressure and 94% a blood lipid measurement in the last 12 months [16]. In comparison to the Swiss study the present study observed 91% of the DM cohort having a blood pressure and 90% a blood cholesterol measurement taken in the last year. Health insurances suggest annual primary care visits and usage of preventive screening such as hemoccult test, colonoscopy, PAP smear and mammography [22]. The recommendation from the Austrian Federal Ministry of Social Affairs, Health, Care and Consumer Protection for annual visits for preventive medical check-ups and gynecologist with PAP smear for women is 18 years, mammography from 45 years on, hemoccult test and colonoscopy from 50 years on [22]. This finding accompanies the beforementioned age recommendation for these procedures. Also, within the DM cohort it was investigated that the > 50 years old DM cohort had a higher frequency of hemoccult test and colonoscopy compared to the < 50 years old DM cohort. Certainly, blood glucose measurements are important for individuals suffering from diabetes mellitus and are carried out at nearly every hospital and doctors’ visit. Further the awareness of the higher mortality of DM individuals with high blood pressure and high blood cholesterol levels led to more frequent preventive care screenings in individuals with DM [23]. More frequent preventive care screening can be influenced by the higher frequency of general practitioner and specialist doctor visits of the DM cohort. Furthermore, more frequent doctor visits could lead to a better communication of the importance of preventive care and give an easier access to preventive care referrals. Next DM individuals are more prone to development of cancer and therefore preventive screening is very important in this specific cohort [24]. Though studies discuss if a higher usage of preventive care, such as mammography, is leading to a higher detection rate of cancer or to false positive cases [25]. In addition, the DM cohort has more comorbidities, which could also be a reason for more preventive care usage and also highlights its importance; however, the non-existing differences in influenza immunization comparing the > 50 years old DM to the > 50 years old Non-DM cohort illustrates that immunization status has to be improved in the vulnerable DM individuals, particularly as this study investigated a higher frequency of general practitioner visits in the > 50 years old DM cohort. Especially, since the vaccination rate against influenza between 2007 and 2014 was decreasing [14]. Therefore, it is crucial to not only understand the immunization usage of DM individuals but also to raise awareness of higher infection morbidity and mortality for them in order to achieve an intact influenza immunization in the whole DM population.

In the present study some limitations have to be reported. The first limitation is the use of self-reported data. Indeed, the usage of self-reported data could lead to overestimated and underestimated frequencies of diseases and care usage. The second limitation is that these data do not differentiate between type 1 and type 2 diabetes mellitus. Due to a worldwide higher prevalence of type 2 diabetes mellitus, we conclude that the majority of this DM cohort are type 2 diabetes mellitus patients [26, 27]. Further, the DM cohort was divided by age and also investigated the DM population over 50 years to make sure reported cases are mostly diabetes mellitus type 2. Lastly, the > 50 years old DM cohort was compared to the > 50 years old Non-DM cohort and the < 50 years old DM cohort.

Novel strength of the present study is that to the best of our knowledge, it is the first to investigate a variety of preventive care screenings, the frequency of different doctor visits and the immunization status in a big DM cohort compared to a non-DM cohort. The findings of the present study could lead to set priorities in order to further improve preventive care in DM individuals, especially for the > 50 years old DM population. Especially, as albeit the higher frequency of doctor visits in the > 50 years old DM population no difference in the frequency of preventive care compared to the > 50 years old Non-DM population is found.

## Supplementary Information

Supplementary tables 1 and 2

## References

[1] Federation, International Diabetes. “Idf Diabetes Atlas” https://diabetesatlas.org/en/.

[2] GBD 2013 Mortality and Causes of Death Collaborators (2015). Global, regional, and national age-sex specific all-cause and cause-specific mortality for 240 causes of death, 1990–2013: a systematic analysis for the global burden of disease study 2013. Lancet.

[3] Roglic G, Unwin N (2010). Mortality attributable to diabetes: estimates for the year 2010. Diabetes Res Clin Pract.

[4] Gregg EW, Zhuo X, Cheng YJ, Albright AL, Narayan KM, Thompson TJ (2014). Trends in lifetime risk and years of life lost due to diabetes in the USA, 1985–2011: a modelling study. Lancet Diabetes Endocrinol.

[5] Litwak L, Goh SY, Hussein Z, Malek R, Prusty V, Khamseh ME (2013). Prevalence of diabetes complications in people with type 2 diabetes mellitus and its association with baseline characteristics in the multinational achieve study. Diabetol Metab Syndr.

[6] Gregg EW, Sattar N, Ali MK (2016). The changing face of diabetes complications. Lancet Diabetes Endocrinol.

[7] Leutner M, Haug N, Bellach L, Dervic E, Kautzky A, Klimek P, Kautzky-Willer A (2021). Risk of typical diabetes-associated complications in different clusters of diabetic patients: analysis of nine risk factors. J Pers Med.

[8] Coughlin SS, Calle EE, Teras LR, Petrelli J, Thun MJ (2004). Diabetes mellitus as a predictor of cancer mortality in a large cohort of us adults. Am J Epidemiol.

[9] Hammer M, Storey S, Hershey DS, Brady VJ, Davis E, Mandolfo N, Bryant AL, Olausson J (2019). Hyperglycemia and cancer: a state-of-the-science review. Oncol Nurs Forum.

[10] Leutner M, Kaleta M, Bellach L, Kautzky A, Thurner S, Klimek P, Kautzky-Willer A (2021). Insulin as monotherapy and in combination with other glucose-lowering drugs is related to increased risk of diagnosis of pneumonia: a longitudinal assessment over two years. JPM.

[11] Ferlita S, Yegiazaryan A, Noori N, Lal G, Nguyen T, To K, Venketaraman V (2019). Type 2 diabetes mellitus and altered immune system leading to susceptibility to pathogens, especially mycobacterium tuberculosis. J Clin Med.

[12] Gupta S, Maratha A, Siednienko J, Natarajan A, Gajanayake T, Hoashi S, Miggin S (2017). Analysis of inflammatory cytokine and Tlr expression levels in type 2 diabetes with complications. Sci Rep.

[13] Pradhan AD, Manson JE, Rifai N, Buring JE, Ridker PM (2001). C-reactive protein, interleukin 6, and risk of developing type 2 diabetes mellitus. JAMA.

[14] Kautzky-Willer A, Dorner T, Jensby A, Rieder A (2012). Women show a closer association between educational level and hypertension or diabetes mellitus than males: a secondary analysis from the Austrian his. BMC Public Health.

[15] Li S, Wang J, Zhang B, Li X, Liu Y (2019). Diabetes mellitus and cause-specific mortality: a population-based study. Diabetes Metab J.

[16] Peytremann-Bridevaux I, Bordet J, Burnand B (2013). Diabetes care in Switzerland: good, but perfectible: a population-based cross-sectional survey. BMC Health Serv Res.

[17] Harris CD, Pan L, Mukhtar Q (2010). Changes in receiving preventive care services among US adults with diabetes, 1997–2007. Prev Chronic Dis.

[18] Luo H, Bell RA, Wright W, Wu Q, Wu B (2018). Trends in annual dental visits among US dentate adults with and without self-reported diabetes and prediabetes, 2004–2014. J Am Dent Assoc.

[19] Myllymäki V, Saxlin T, Knuuttila M, Rajala U, Keinänen-Kiukaanniemi S, Anttila S, Ylöstalo P (2018). Association between periodontal condition and the development of type 2 diabetes mellitus-results from a 15-year follow-up study. J Clin Periodontol.

[20] Michaud DS, Fu Z, Shi J, Chung M (2017). Periodontal disease, tooth loss, and cancer risk. Epidemiol Rev.

[21] Borgnakke WS, Ylöstalo PV, Taylor GW, Genco RJ (2013). Effect of periodontal disease on diabetes: systematic review of epidemiologic observational evidence. J Periodontol.

[22] Österreich, Öffentliches Gesundheitsportal. “Vorsorge.” https://www.gesundheit.gv.at.

[23] Stamler J, Vaccaro O, Neaton JD, Wentworth D (1993). Diabetes, other risk factors, and 12-yr cardiovascular mortality for men screened in the multiple risk factor intervention trial. Diabetes Care.

[24] Suh S, Kim KW (2019). Diabetes and cancer: cancer should be screened in routine diabetes assessment. Diabetes Metab J.

[25] Calip GS, Yu O, Boudreau DM, Shao H, Oratz R, Richardson SB, Gold HT (2019). Diabetes and differences in detection of incident invasive breast cancer. Cancer Causes Control.

[26] Lascar N, Brown J, Pattison H, Barnett AH, Bailey CJ, Bellary S (2018). Type 2 diabetes in adolescents and young adults. Lancet Diabetes Endocrinol.

[27] Maahs DM, West NA, Lawrence JM, Mayer-Davis EJ (2010). Epidemiology of type 1 diabetes. Endocrinol Metab Clin North Am.

