# Supplementary material for: Need for improving immunization status and preventive care in diabetes mellitus patients
Source: Wien Klin Wochenschr. 2022 Sep 22;135(13-14):336–42. doi: 10.1007/s00508-022-02080-5 (PMC10338608; doi:10.1007/s00508-022-02080-5)
Supplement: Supplementary file 1 — Supplementary tables 1 and 2 [file 508_2022_2080_MOESM1_ESM.docx]

Supplemental material

Table 1: Frequencies of preventive care for the DM and non-DM cohort and all p-values for the linear regression analyses. Frequencies preventive care for the over 50 years DM and the over 50 years Non-DM cohort and all p-values for the linear regression analyses.

| **Variable name** | **DM (n=678)** | **Non-DM (n=15,093)** | **p-value** | **Over 50 years DM (n=592)** | **Over 50 years Non-DM (n=6,689)** | **p-value** |
| --- | --- | --- | --- | --- | --- | --- |
| Screening of blood cholesterol  -in the last 12 months  -in the last 1-3 years  -in the last 3-5 years  -over 5 years ago  -never | 610 (90,0%)  51 (7,5%)  5 (0,7%)  6 (0,9%)  6 (0,9%) | 8,425 (55,8%)  3,951 (26,2%)  856 (5,7%)  766 (5,1%)  1,095 (7,3%) | <0,001 | 540 (90,0%)  37 (7,5%)  5 (0,7%)  4 (0,9%)  6 (0,9%) | 4,595 (68,7%)  1,459 (21,8%)  259 (3,9%)  226 (3,4%)  150 (2,2%) | <0,001 |
| Screening of blood glucose  -in the last 12 months  -in the last 1-3 years  -in the last 3-5 years  -over 5 years ago  -never | 625 (92,2%)  47 (7,0%)  2 (0,3%)  3 (0,4%)  1 (0,1%) | 8,633 (57,2%)  3,875 (25,7%)  849 (5,6%)  809 (5,4%)  927 (6,1%) | <0,001 | 552 (91,2%)  35 (5,9%)  1 (0,2%)  3 (0,5%)  1 (0,2%) | 4,619 (69,1%)  1,390 (20,8%)  271 (4,1%)  213 (3,9%)  196 (2,9%) | <0,001 |
| Last blood pressure measurement  -in the last 12 months  -in the last 1-3 years  -in the last 3-5 years  -over 5 years ago  -never | 617 (91,0%)  52 (7,7%)  4 (0,6%)  3 (0,4%)  2 (0,3%) | 10,334 (68,5%)  3,324 (22,0%)  698 (4,6%)  566 (3,8%)  171 (1,1%) | <0,001 | 544 (91,9%)  40 (6,8%)  3 (0,5%)  3 (0,5%)  2 (0,3%) | 5,156 (77,1%)  1,076 (16,1%)  227 (3,3%)  183 (2,7%)  47 (0,7%) | <0,001 |
| Last test for occult blood in feces  -in the last 12 months  -in the last 1-2 years  -in the last 2-3 years  -over 3 years ago  -never | 221 (32,6%)  116 (17,1%)  47 (6,9%)  140 (20,6%)  154 (22,7%) | 3,335 (22,1%)  1,970 (13,1%)  1,125 (7,5%)  2,708 (18,0%)  5,955 (39,5%) | <0,001 | 202 (34,1%)  106 (17,9%)  43 (7,3%)  129 (21,8%)  112 (18,9%) | 2,225 (33,3%)  1,141 (17,1%)  666 (10,0%)  1,251 (18,7%)  1,406 (21,2%) | >1 |
| Last colonoscopy  -in the last 12 months  -in the last 1-5 years  -in the last 5-10 years  -over 10 years ago  -never | 76 (11,2%)  206 (30,4%)  67 (10,0%)  37 (5,5%)  292 (43,1%) | 1,027 (6,8%)  2,655 (17,6%)  1,065 (7,1%)  607 (4,0%)  9,379 (62,1%) | <0,001 | 70 (11,8%)  200 (33,8%)  60 (10,1%)  34 (5,7%)  228 (38,5%) | 796 (11,9%)  2,011 (30,1%)  717 (10,7%)  348 (5,2%)  2,871 (42,9%) | >1 |
| Last mammography  -in the last 12 months  -in the last 1-2 years  -in the last 2-3 years  -over 3 years ago  -never | (women=321)  117 (36,4%)  73 (22,7%)  30 (9,3%)  63 (19,6%)  38 (11,8%) | (women=8,465)  2,621 (31,0%)  1,624 (19,2%)  761 (9,0%)  1,006 (11,9%)  2,462 (28,7%) | <0,001 | (women=279)  107 (38,4%)  67 (24,0%)  26 (9,3%)  58 (20,1%)  21 (7,5%) | (women=3,771)  1,623 (43,0%)  947 (25,1%)  427 (11,3%)  592 (15,7%)  182 (4,8%) | 0,01 |
| Last PAP smear  -in the last 12 months  -in the last 1-2 years  -in the last 2-3 years  -over 3 years ago  -never | (women=321)  134 (41,7%)  59 (18,4%)  17 (5,3%)  87 (27,1%)  24 (7,5%) | (women=8,465)  5,018 (59,3%)  1,619 (19,1%)  495 (5,8%)  937 (11,1%)  396 (4,7%) | <0,05 | (women=279)  110 (39,4%)  52 (18,6%)  15 (5,8%)  84 (30,1%)  18 (6,5%) | (women=3,771)  1,850 (49,1%)  715 (19,0%)  292 (7,7%)  735 (19,5%)  143 (3,8%) | 0,05 |

*DM* diabetes mellitus*, Non-DM* non-diabetes mellitus*, PAP* Papanicolaou

Table 2: Frequencies of doctors’ visits, preventive care and immunization status for the over 50 years and under 50 years DM cohort

| **Variable name** | **Over 50 years DM (n=592)** | **Under 50 years DM (n=86)** | **p-value** |
| --- | --- | --- | --- |
| Intact immunization against tetanus | 401 (67,7%) | 74 (86,0%) | <0,001 |
| Intact immunization against diphtheria | 214 (36,1%) | 55 (64,0%) | <0,001 |
| Intact immunization against Polio | 205 (34,6%) | 46 (53,5%) | <0,001 |
| Intact immunization against pneumococcus | 56 (13,8%) | 333 (9,9%) | <0,001 |
| Intact immunization against influenza | 90 (13,3%) | 1,072 (7,1%) | <0,001 |
| Intact immunization against TBE | 396 (66,9%) | 58 (67,4%) | <0,001 |
| Hospitalized in the last 12 months | 190 (32,1%) | 14 (16,3%) | <0,001 |
| Dentist  -in the last 6 months  -in the last 6-12 months  -over 12 months ago | 225 (38,0%)  121 (20,4%)  246 (41,5%) | 38 (44,2%)  25 (29,1%)  23 (26,7%) | 0,01 |
| General practitioner  -in the last 12 months  -over 12 months ago  -never | 534 (90,2%)  55 (9,3%)  3 (0,5%) | 72 (83,7%)  14 (16,3%)  0 (0,0%) | >1 |
| Specialist doctor  -in the last 12 months  -over 12 months ago  -never | 436 (73,6%)  146 (24,7%)  10 (1,7%) | 69 (80,2%)  17 (19,8%)  0 (0,0%) | 0,05 |
| Screening of blood cholesterol  -in the last 12 months  -in the last 1-3 years  -in the last 3-5 years  -over 5 years ago  -never | 540 (90,0%)  37 (7,5%)  5 (0,7%)  4 (0,9%)  6 (0,9%) | 70 (90,0%)  14 (7,5%)  1 (0,7%)  0 (0,0%)  0 (0,0%) | >1 |
| Screening of blood glucose  -in the last 12 months  -in the last 1-3 years  -in the last 3-5 years  -over 5 years ago  -never | 552 (91,2%)  35 (5,9%)  1 (0,2%)  3 (0,5%)  1 (0,2%) | 73 (81,4%)  12 (14,0%)  1 (1,2%)  0 (0,0%)  0 (0,0%) | 0,1 |
| Last blood pressure measurement  -in the last 12 months  -in the last 1-3 years  -in the last 3-5 years  -over 5 years ago  -never | 544 (91,9%)  40 (6,8%)  3 (0,5%)  3 (0,5%)  2 (0,3%) | 73 (84,5%)  12 (14,0%)  1 (1,2%)  3 (3,5%)  2 (2,3%) | 0,05 |
| Last test for occult blood in feces  -in the last 12 months  -in the last 1-2 years  -in the last 2-3 years  -over 3 years ago  -never | 202 (34,1%)  106 (17,9%)  43 (7,3%)  129 (21,8%)  112 (18,9%) | 19 (22,1%)  10 (11,6%)  4 (4,7%)  11 (12,8%)  42 (48,8%) | <0,001 |
| Last colonoscopy  -in the last 12 months  -in the last 1-5 years  -in the last 5-10 years  -over 10 years ago  -never | 70 (11,8%)  200 (33,8%)  60 (10,1%)  34 (5,7%)  228 (38,5%) | 6 (7,0%)  6 (7,0%)  7 (8,1%)  3 (3,4%)  64 (74,4%) | <0,001 |
| Last mammography  -in the last 12 months  -in the last 1-2 years  -in the last 2-3 years  -over 3 years ago  -never | (female=279)  107 (38,4%)  67 (24,0%)  26 (9,3%)  58 (20,1%)  21 (7,5%) | (female=42)  10 (23,8%)  6 (14,3%)  4 (9,5%)  5 (11,9%)  17 (40,5%) | >1 |
| Last PAP smear  -in the last 12 months  -in the last 1-2 years  -in the last 2-3 years  -over 3 years ago  -never | (female=279)  110 (39,4%)  52 (18,6%)  15 (5,8%)  84 (30,1%)  18 (6,5%) | (female=42)  24 (57,1%)  7 (16,7%)  2 (4,8%)  3 (7,1%)  6 (14,3%) | N.S. |

*DM* diabetes mellitus, *TBE* tick-borne encephalitis, *PAP* Papanicolaou
